# 

**DOI:** 10.1192/bjb.2023.96

**Published:** 2024-08

**Authors:** Sukhmeet Singh

**Affiliations:** Dual trainee in Child and Adolescent and Intellectual Disabilities Psychiatry, NHS Greater Glasgow and Clyde, Glasgow, UK. Email: singhsu@nhs.scot

**Figure:**
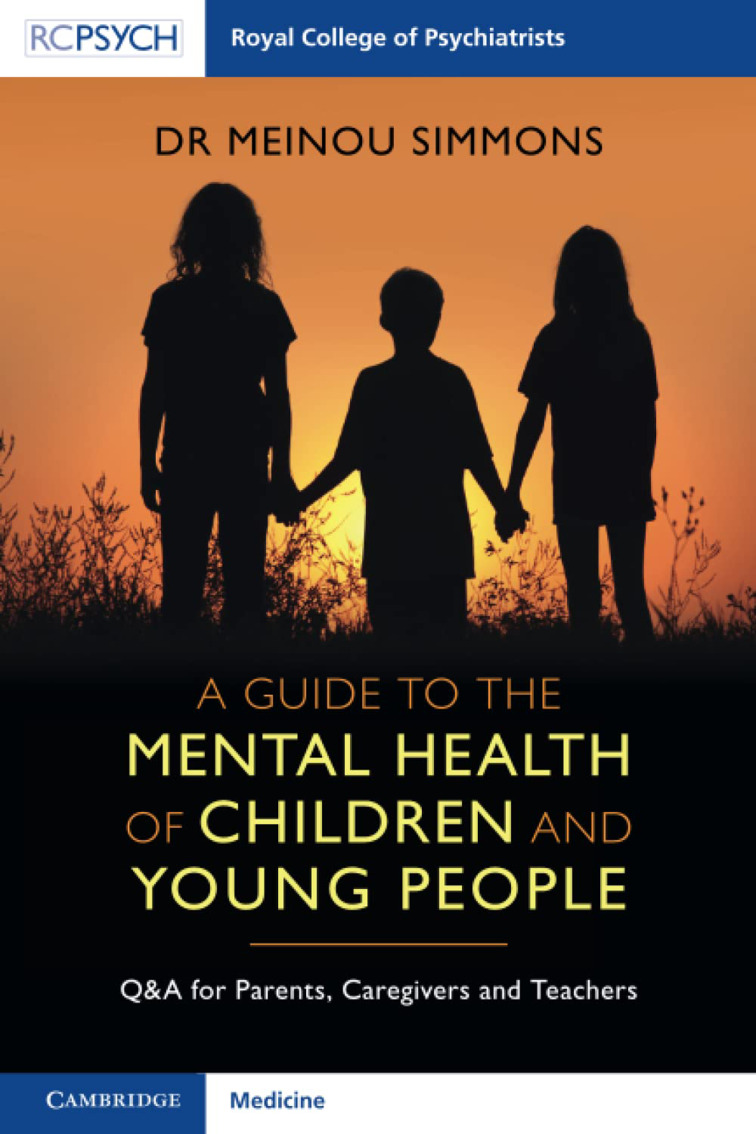

In recent years there has been an explosion in the concern raised about the mental health and well-being of children and young people. Services are overwhelmed with parents, caregivers and educators struggling to find reliable information. The COVID-19 pandemic further accentuated the focus on children's and young people's mental health because of the difficulties they experienced in accessing educational and social opportunities. In this book Dr Simmons aims to combat misinformation by providing the general public with reliable information and a guide to further reading to help them to look after children and young people.

The book is split into three parts. The first part considers factors affecting children's and young people's mental health, including a section on managing the impact of the coronavirus pandemic as well as potentially more difficult areas for parents and teachers, such as the role of sex and romantic relationships and experiences of discrimination in the mental well-being of young people. The second part focuses on how to strengthen relationships with children and young people and provide support. This includes a discussion about how to support co-parents, such as grandparents, as well as positive parenting strategies. The third part comprises chapters offering information on the common mental health problems experienced by children and young people.

The book is accessible, with an excellent format that makes it easy to find information. The language is clear, precise and avoids jargon. The links to further reading are wide-ranging and helpful. The book includes a helpful discussion about the value of using diagnoses in children and young people which will also be of benefit to clinicians. The chapters on individual disorders present an excellent summary with reference to useful case vignettes.

There are, however, areas that could have been considered for inclusion, such as the cross-cultural aspects of mental health in children and young people, including the impact of experiences of migration and being a child or young person who does not have English as their first language. Naturally, the information available is continually evolving and this type of book may require continuous updates to prevent it becoming outdated. Nevertheless, the book provides very useful information for many groups, including clinicians.

